# Association between the No-Reflow Phenomenon and Soluble CD40 Ligand Level in Patients with Acute ST-Segment Elevation Myocardial Infarction

**DOI:** 10.3390/medicina55070376

**Published:** 2019-07-15

**Authors:** Mustafa Begenc Tascanov, Zulkif Tanriverdi, Fatih Gungoren, Feyzullah Besli, Muslihittin Emre Erkus, Ataman Gonel, Ismail Koyuncu, Recep Demirbag

**Affiliations:** 1 Department of Cardiology, Harran University, Faculty of Medicine, Sanlıurfa, 63300, Turkey; 2 Department of Medical Biochemistry, Harran University, Faculty of Medicine, Sanlıurfa 63300, Turkey

**Keywords:** no-reflow phenomenon, sCD40L, ST-elevation myocardial infarction

## Abstract

*Background and objectives*: No-reflow (NR) phenomenon is defined as insufficient myocardial perfusion in coronary circulation in the absence of angiographic evidence of mechanical obstruction. The primary mechanisms of the NR occurrence are thought to be high platelet activity and thrombus burden. Soluble CD40 ligand (sCD40L), which is released into the plasma following platelet activation, accelerates the inflammatory process and causes further platelet activation. The aim of our study is to investigate the relationship between the NR phenomenon and sCD40L level in patients with ST-elevation myocardial infarction (STEMI). *Methods:* A total of 81 acute STEMI patients undergoing primary percutaneous coronary intervention and 40 healthy participants were included in this study. Acute STEMI patients were classified into two groups: 41 patients with the NR phenomenon (NR group) and 40 patients without the NR phenomenon (non-NR group). The serum sCD40L level was measured for all groups. *Results:* The serum sCD40L level was significantly higher in the NR group than in non-NR and control groups (379 ± 20 pg/mL, 200 ± 15 pg/mL and 108 ± 6.53 pg/mL, respectively; *p* < 0.001). Univariate regression analysis demonstrated that male sex, age, Gensini score and sCD40L level were the possible factors affecting the occurrence of the NR phenomenon. In multivariate regression analysis, age (odds ratio [OR], 1.091; 95% confidence interval [CI], 1.023–1.163; *p* < 0.008) and serum sCD40L (OR, 1.016; 95% CI, 1.008–1.024; *p* < 0.001) remained the independent predictor of the presence of NR. *Conclusions:* Our study showed that serum sCD40L level was an independent predictor of the NR phenomenon occurrence.

## 1. Introduction

Acute myocardial infarction (AMI) is one of the most common causes of death worldwide [1]. ST-elevation myocardial infarction (STEMI), the most severe clinical manifestation of AMI, is a critical clinical condition requiring early diagnosis and emergency treatment. The preferred treatment modality to achieve vessel patency in STEMI is primary percutaneous coronary intervention (pPCI) [2]. One of the most important problems affecting the success of pPCI in patients with STEMI is the no-reflow (NR) phenomenon. The fact that the NR phenomenon is a strong predictor of major cardiovascular events, such as malignant arrhythmia, cardiac death and congestive heart failure, highlights its importance [3,4,5]. No-reflow phenomenon is defined as insufficient myocardial perfusion in coronary circulation in the absence of angiographic evidence of mechanical obstruction [6]. The distal atherothrombotic embolism, endothelial injury, ischemic injury, reperfusion injury and vasospasm may occur as a result of local platelet activation [7]. Although the exact mechanism of the NR occurrence has not been completely understood, the primary mechanism is thought to be increased platelet activation. CD40 ligand (CD40L) is a transmembrane protein belonging to the TNF receptor superfamily. It is usually found in platelets and is released into the plasma following platelet activation; thereafter it is transformed into soluble CD40L (sCD40L). The interaction of sCD40L with CD40 on the endothelium triggers the release of inflammatory cytokines, activates matrix metalloproteinases and boosts the coagulation cascade, leading to further platelet activation [8]. There is no study investigating the relationship between NR phenomenon and sCD40L levels in patients with STEMI undergoing pPCI. The aim of our study is to evaluate the relationship between the NR phenomenon and sCD40L level in patients with STEMI undergoing pPCI.

## 2. Materials and Methods

### 2.1. Study Population

This study was designed as a prospective case-control study. 81 acute STEMI patients undergoing primary percutaneous coronary intervention and 40 healthy participants were included in this study. Acute STEMI patients were classified into two groups: 41 patients with the NR phenomenon (NR group) and 40 patients without the NR phenomenon (non-NR group). Patients with previous known coronary artery disease, heart failure, severe heart valve disease, malignancy, hematological and rheumatic diseases, renal failure and liver disease; and those who did not provide consent for participation in the study were excluded. The study was approved by the Harran University, Faculty of Medicine Ethics Committee (number: 74059997-050.04.04, issued on 06.09.2018.) and was implemented in accordance with the rules of the Declaration of Helsinki. Informed consent was obtained from all patients included in the study. The diagnosis of STEMI was confirmed based on the definition of the fourth universal myocardial infarction, and treatment was managed according to the American Heart Association (AHA) guidelines [9,10]. The NR phenomenon was defined as thrombolysis in myocardial infarction (TIMI) <2 coronary blood flow according to the current TIMI rating without dissection and stenosis [11]. Gensini score was used to evaluate the coronary artery severity index [12].

### 2.2. Primary Percutaneous Coronary Intervention

All study participants underwent coronary angiography performed projections using the Judkins technique. pPCI procedure was performed using the standard radial route with 6F guiding catheters or using femoral route with 6F or 7F. All angiographic data was analyzed by two independent cardiologists. The inter-observer and intra-observer concordance rate for no-reflow phenomenon were 98.5% and 99%, respectively. Before the procedure, all patients were administered 180 mg of ticagrelor with 300 mg of aspirin, and intravenous unfractionated heparin was administered as a 70 U/kg bolus. Additional heparin was administered at active coagulation time (ACT) >250 to provide adequate anticoagulation during the procedure. The drug-eluting stent was placed into the infarct related artery for the known technique. Patients in the NR group were treated according to the recommendations of current guidelines. Glycoprotein IIb/IIIa inhibitors and intracoronary vasodilator agents was used depending operator’s preference.

### 2.3. Biochemical Analysis and Serum sCD40L Assay

Blood samples were collected for the evaluation of laboratory parameters in the emergency department after ECG was taken during the first evaluation of patients without any drug and additional treatment. The blood samples were centrifuged for 10 min at 3000 rpm, and the serum was stored at −80°C in aliquots until the day of analysis. The samples were analyzed using standard laboratory methods to determine blood glucose, electrolytes, total cholesterol, high-density lipoprotein, low-density lipoprotein and triglyceride levels. Serum sCD40L levels were measured using the enzyme-linked immunosorbent assay according to the manufacturer’s indications (Elabscience, Houston, TX, USA).

### 2.4. Statistical Analyses

The statistical package for the social sciences software (SPSS 20.0 for Windows, Chicago, IL, USA) was used for all statistical analysis. The data were tested for normal distribution using the Kolmogorov–Smirnov test. Continuous variables were expressed as means ± SD. Student t test was used for the two-group comparisons, and the one-way ANOVA test was used for the three-group comparisons. Chi square test was performed for the comparison of categorical variables. Correlation analysis was performed to determine correlation of sCD40L with other continuous variables. Variables having an unadjusted P value less than 0.1 in univariate analysis were considered as potential risk factors of NR occurrence and included in the full multivariate regression model. Multivariate logistic regression analysis was used to determine the independent predictor of the NR occurrence. Receiver operating characteristic (ROC) curve analysis was used to determine the optimum cut-off value of sCD40L level for predicting the NR occurrence after pPCI in patients with STEMI. A *p*-value of <0.05 was considered statistically significant.

## 3. Results

**Figure 1:**
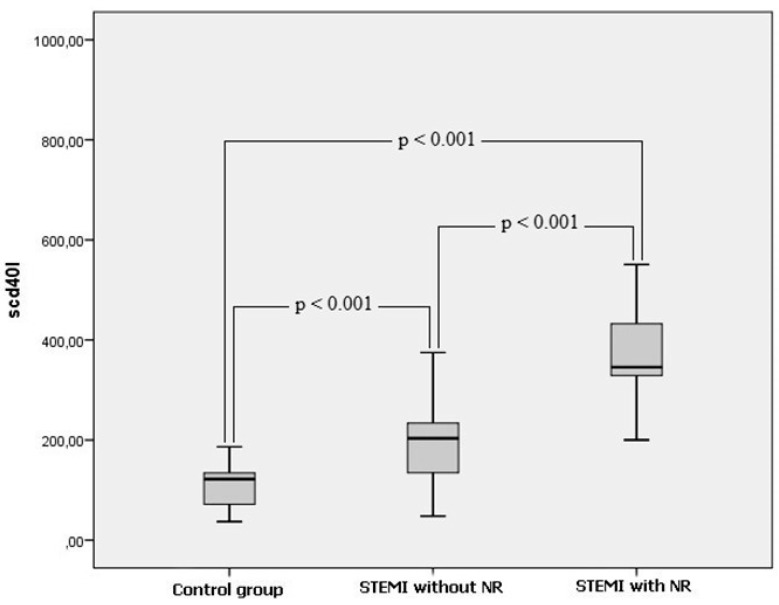
Comparison of s CD40L soluble CD40 ligand level among the three groups.

**Figure 2:**
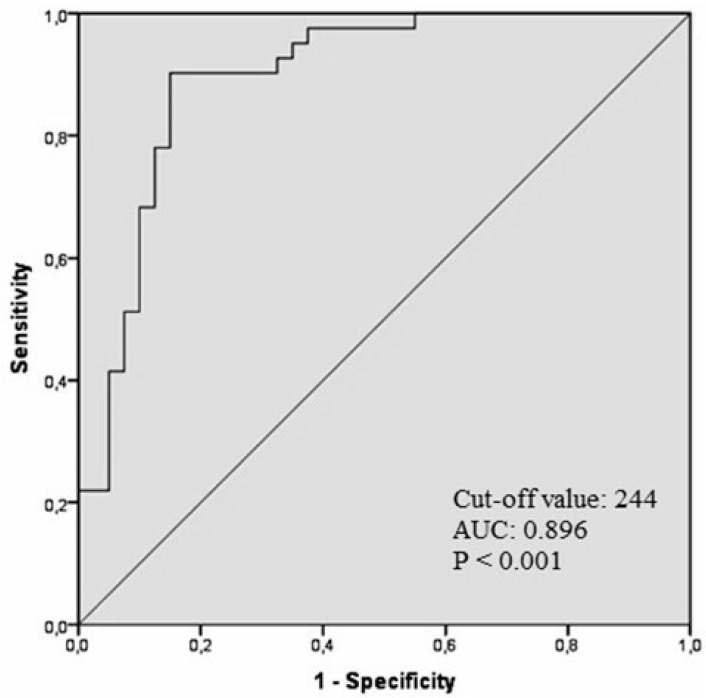
ROC Reciever operator characteristics curve analysis of soluble CD40 ligand and the no-reflow phenomenon.

Clinical characteristics of the study population were presented in Table 1. There was no significant difference among the groups in terms of sex, diabetes, hypertension, smoking and body mass index (*p* > 0.05, for all). However, significant difference existed among the groups for systolic blood pressure, age, and Gensini score (*p* = 0.001, *p* = 0.001 and *p* = 0.003, respectively). Laboratory parameters of study groups were listed in Table 2. Admission serum sCD40L, WBC and ESR (*p* = 0.001, *p* = 0.001 and *p* = 0.003, respectively) were significantly different among the three groups. Three groups were compared with each other to better elucidate the clinical importance of sCD40L. STEMI patients with NR had significantly higher level of sCD40L than patients without NR and control group. In addition, STEMI patients without NR had significantly higher level of sCD40L than control group (Figure 1). In correlation analysis, sCD40L was positively correlated with age (*r* = 0.191, *p* = 0.036), Gensini score (*r* = 0.343, *p* = 0.002), ESR (*r* = 0.386, *p* < 0.001) and WBC (*r* = 0.378, *p* < 0.001), whereas sCD40L negatively correlated with systolic blood pressure (*r* = −0.345, *p* < 0.001). Univariate regression analysis revealed that age, male gender, Gensini score and sCD40L levels were possibly factors responsible for the NR occurrence. Multivariate logistic regression analysis showed that age (odds ratio [OR], 1.091; 95% confidence interval [CI], 1.023–1.163; *p* < 0.008) and serum sCD40L levels (OR, 1.016; 95% CI, 1.008–1.024; *p* < 0.001) were the independent predictors of the NR occurrence (Table 3). ROC curve analysis was used to determine the optimal cut-off value of sCD40L for predicting the NR occurrence after primary PCI in patients with STEMI. sCD40L ≥ 244 pg/mL predicted the NR occurrence with a sensitivity of 85% and specificity of 90% (area under curve: 0.896, 95% CI: 0.825–0.968, *p* < 0.001) (Figure 2).

## 4. Discussion

In the present study, we found that sCD40L level was strongly correlated with the NR occurrence in patients undergoing pPCI for STEMI. To our knowledge, this is the first study demonstrating the independent relationship between the sCD40L and NR occurrence.

Acute coronary syndrome begins with platelet activation and thrombus formation after plaque erosion or rupture. Antiplatelet therapy prevents platelet activation; therefore it is the most critical treatment in ACS [13,14,15]. Although the exact mechanism of the NR occurrence has not been completely understood, the primary mechanism is thought to be increased platelet activation. Previous studies reported that the frequency of NR occurrence in acute STEMI after pPCI ranges from 2.3% to 12% [3,16,17]. However, our study does not give any idea about this topic due to it being a case-control study.

sCD40L contributes to the pathophysiology of atherosclerosis and atherothrombosis [18]. Because of its autocrine, endocrine, paracrine activities, sCD40L increases platelet activation, aggregation and platelet-leukocyte conjugation, which may lead to atherothrombosis [8]. Previous studies revealed that sCD40L significantly increased in patients with diabetes mellitus and hypercholesterolemia in whom coronary artery disease was proven [19,20]. The fact that high levels of sCD40L in ACS demonstrate that it plays a major role in triggering atherothrombotic events [21,22]. C7E3 Fab Antiplatelet Therapy in Unstable Refractory Angina (CAPTURE) and oral glycoprotein IIb/IIIa inhibition with orbofiban in patients with unstable coronary syndromes (OPUS-TIMI16) studies showed that increased sCD40L levels were associated with a higher risk for death and non-fatal myocardial infarction [23,24]. In addition, Li et al. reported that elevated sCD40L level was an independent predictor of recurrent stroke in patients with transient ischemic attack and small stroke [25]. Consistent with the literature, we also demonstrated that sCD40L was significantly higher in patients with acute STEMI compared to the control group. These findings suggest that an inflammatory process is triggered in acute STEMI. However, in addition to this finding, we detected that sCD40L was significantly higher in the NR group than in the non-NR. Moreover, sCD40L was independent predictors of the NR occurrence and sCD40L ≥244 pg/mL predicted the NR occurrence with a sensitivity of 85% and specificity of 90%. It can be concluded NR phenomenon occurs more frequent in acute STEMI patients who has a more intense inflammatory process.

Inflammation plays a crucial role in the development of atherosclerosis [26]. Activation of inflammatory cells may enhance NR phenomenon. Previous studies showed that inflammatory markers are important factors for predicting the NR occurrence [27,28]. In this study, we evaluated WBC, hs-CRP and ESR as inflammatory markers. We found that these parameters were significantly higher in STEMI patients with NR compared to STEMI patients without NR. However, when we included all these inflammatory markers and sCD40L to regression analyses, we found that sCD40L was the only independent predictor of NR occurrence in acute STEMI patients. These findings suggest that sCD40L has higher clinical importance than others inflammatory markers for predicting the NR occurrence in acute STEMI patients.

No-reflow occurrence is related to poor prognostic events in acute STEMI [3,29]. Also, the Gensini score, which reflects the severity and prevalence of coronary atherosclerosis, is a prognostic marker associated with poor cardiac outcomes [30]. In our study, we found that the Gensini score was significantly higher in acute STEMI patients with NR than in acute STEMI patients without NR. Moreover, sCD40L was positively correlated with the Gensini score. Therefore, it may be assumed that the severity of coronary artery disease predicts the NR occurrence. However, the Gensini score was not an independent predictor of NR occurrence in multivariate analysis in our study. This may be because our study used a small number of patients. In addition to the Gensini score, age is also strongly associated with the NR occurrence. Harrison et al. showed that age was an independent predictor of the NR phenomenon occurrence in patients with STEMI [16]. Similar to this study, we also detected that age was the independent predictor of the NR occurrence in acute STEMI.

Our study has some limitations. First, the most significant limitations of our study were the small sample size and single-center nature. Second, we evaluated only baseline sCD40L levels and did not perform serial sCD40L measurements after pPCI. It could be useful to measure sCD40L after pPCI in NR and non-NR group. Third, we did not evaluate hospital and long-term mortality. It could be more interesting to investigate the association of sCD40L with the prognosis of acute STEMI. Fourth, the procedure-related data Syntax score was not calculated in this study. However, we investigated the Gensini score, which is a marker of coronary artery disease severity. Further prospective studies with larger participant are required for better determining the relationship between sCD40L and NR phenomenon.

## 5. Conclusions

No-reflow occurrence is associated with poor prognosis in acute STEMI. In this study, we have for the first time shown that high serum sCD40L levels may independently predict the occurrence of NR after pPCI in acute STEMI patients.

## Figures and Tables

**Table 1:** Baseline characteristics of the study population.

Variables	STEMI with NR (*n* = 41)	STEMI without NR (*n* = 40)	Control Group (*n* = 40)	*P*
Age (years)	64 ± 8	54 ± 11	59 ± 10	0.001
Male gender (%)	31 (75.6)	21 (52.5)	25 (62.5)	0.095
SBP (mmHg)	115 ± 12	125 ± 10	126 ± 10	0.001
DBP (mmHg)	75 ± 8	77 ± 9	78 ± 9	0.07
Smoking (%)	18 (43.9)	16 (40)	15 (37.5)	0.84
Hypertension (%)	13 (31.7)	11 (27.5)	12 (30)	0.92
Diabetes mellitus (%)	14 (35)	12 (30)	10 (25)	0.67
Heart rate on admission (/beat)	71 ± 10	67 ± 8	65 ± 7	0.62
Body mass index (kg/m^2^)	26 ± 5.1	26 ± 4.4	27 ± 4.3	0.60
Gensini score	38 ± 3.3	35 ± 3.1	-	0.003
Stent length (mm)	28 ± 8	27 ± 7	-	0.65

SBP: systolic blood pressure; DBP: diastolic blood pressure; NR: no-reflow; STEMI: ST elevated myocardial infarction; pPCI: primary percutaneous coronary intervention; mm: millimeter.

**Table 2:** Baseline laboratory measurements of the study population.

Variables	STEMI with NR (*n* = 41)	STEMI without NR (*n* = 40)	Control group (*n* = 40)	*P*
Glucose (mg/dL)	88 ± 7.2	91 ± 6.3	88 ± 6.4	0.72
Urea (mg/dL)	31 ± 13	30 ± 12	30 ± 17	0.20
Creatinine (mg/dL)	1.0 ± 0.4	0.9 ± 0.2	1.0 ± 0.3	0.20
Hemoglobin (g/dL)	13 ± 1.8	14 ± 1.7	13 ± 1.6	0.90
WBC (103/µL)	13 ± 3.1	12 ± 3.2	11 ± 2.6	0.001
Platelets (103/µL)	265 ± 78	262 ± 57	247 ± 57	0.78
Total cholesterol (mg/dL)	169 ± 43	181 ± 56	183 ± 64	0.74
Triglyceride (mg/dL)	120 ± 13	125 ± 17	135 ± 21	0.83
HDL cholesterol (mg/dL)	41 ± 10	44 ± 18	43 ± 17	0.31
LDL cholesterol (mg/dL)	108 ± 41	112 ± 42	113 ± 42	0.70
hs-CRP (mg/dL)	2.9 ± 3.2	5.1 ± 3	1 ± 2	0.50
ESR (mm/h)	25 ± 17	20 ± 16	5 ± 3	0.001
sCD40L (pg/mL)	339 ± 19	200 ± 15	108 ± 6	0.001

HDL: high-density lipoprotein; LDL: low density lipoprotein; WBC: white blood count; hs-CRP: high sensitive C reactive protein; NR: no-reflow; STEMI: ST elevated myocardial infarction; ESR: erythrocyte sedimentation rate.

**Table 3:** Univariate and multivariate logistic regression analysis representing the independent predictors of NR phenomenon.

	Univariate		Multivariate	
Variables	OR (95% CI)	*P*	OR (95% CI)	*P*
Age	1.118 (1.057–1.183)	0.001	1.091 (1.023–1.163)	0.008
Male gender	2.805(1.090–7.217)	0.032	3.978(0.841–18.816)	0.082
sCD40L	1.019 (1.011–1.026)	0.001	1.016 (1.008–1.024)	0.001
Gensini	0.831 (1.039–1.410)	0.014	1.142(0.947–1.450)	0.146
Hypertension	1.221(0.470–3.185)	0.679		
Diabetes mellitus	1.210(0.475–3.81)	0.690		
Stent length	1.018 (0.957–1.082)	0.574		
Platelets	0.996 (0.990–1.003)	0.245		
WBC	0.950 (0.827–1.092)	0.470		
hs-CRP	0.928 (0.845–1.019)	0.118		
ESR	1.016(0.989–1.043)	0.249		

sCD40L: soluble CD40 ligand; WBC: white blood count; hs-CRP: high sensitive C reactive protein; ESR: erythrocyte sedimentation rate.
